# The sight of an adult brood parasite near the nest is an insufficient cue for a honeyguide host to reject foreign eggs

**DOI:** 10.1111/ibi.12254

**Published:** 2015-04-11

**Authors:** Wenfei Tong, Nicholas P C Horrocks, Claire N Spottiswoode

**Affiliations:** 1 Department of Zoology, University of CambridgeDowning Street, Cambridge, CB2 3EJ, UK; 2 Percy FitzPatrick Institute, DST-NRF Centre of Excellence, University of Cape TownRondebosch, 7701, South Africa

**Keywords:** brood parasitism, egg rejection, frontline defences, mobbing

## Abstract

Hosts of brood-parasitic birds typically evolve anti-parasitism defences, including mobbing of parasitic intruders at the nest and the ability to recognize and reject foreign eggs from their clutches. The Greater Honeyguide *Indicator indicator* is a virulent brood parasite that punctures host eggs and kills host young, and accordingly, a common host, the Little Bee-eater *Merops pusillus* frequently rejects entire clutches that have been parasitized. We predicted that given the high costs of accidentally rejecting an entire clutch, and that the experimental addition of a foreign egg is insufficient to induce this defence, Bee-eaters require the sight of an adult parasite near the nest as an additional cue for parasitism before they reject a clutch. We found that many Little Bee-eater parents mobbed Greater Honeyguide dummies while ignoring barbet control dummies, showing that they recognized them as a threat. Surprisingly, however, neither a dummy Honeyguide nor the presence of a foreign egg, either separately or in combination, was sufficient to stimulate egg rejection.

Host defences against brood parasitism are most effective if they occur before a brood parasite enters the nest (Kilner & Langmore 2011, Feeney *et al*. 2012): if a host can hinder a female brood parasite by mobbing her, it reduces the risk of host eggs being damaged or removed during parasitic laying (Gloag *et al*. 2013). Moreover, if a host sees a female brood parasite near its nest, it can use this information to refine subsequent egg rejection behaviour and reduce the risk of misidentifying and mistakenly rejecting one of its own eggs (Davies *et al*. 1996). We should therefore expect frontline defences such as nest guarding and mobbing to be particularly prevalent in the hosts of virulent brood parasites that, by monopolizing and extending host parental care, impose the greatest fitness costs on their hosts.

The Greater Honeyguide *Indicator indicator* is one such parasite. Female Honeyguides often puncture all the eggs in a nest they parasitize (Spottiswoode & Colebrook-Robjent 2007). Consequently, hosts that fail to deter a Honeyguide stand to lose all their current reproductive investment regardless of whether the parasitic egg hatches. Any host chicks hatching from eggs that escape puncturing, or hatching despite being punctured, are rapidly killed by the young Honeyguide using its specially adapted bill hook (Spottiswoode & Koorevaar 2012). Host parents then spend over a month raising the parasitic chick, removing any possibility of re-nesting in the same season. Honeyguide parasitism is thus very costly to hosts, and we should expect selection to have favoured strong anti-parasitic defences in frequently parasitized species. In our study population, the most heavily parasitized host is the Little Bee-eater *Merops pusillus*: on average 65.7% of nests are visited by a Greater Honeyguide (Spottiswoode & Koorevaar 2012). However, Little Bee-eaters subsequently desert just over half of these, contributing to an overall rate of successful parasitism of 28.5% (Spottiswoode & Koorevaar 2012). Given such strong selection pressure from brood parasites, it is puzzling that the experimental addition of a foreign egg to Bee-eater nests is an insufficient cue to trigger defensive clutch desertion or egg rejection (Spottiswoode 2013).

A possible explanation is that unlike in most other brood parasitic systems, rejecting the foreign egg alone is an insufficient defence. This is because Honeyguides puncture host eggs, preventing a parasitized clutch from hatching even if the Honeyguide fails to lay a viable egg, or if hosts reject the parasitic egg. An adaptive response by bee-eaters is to reject entire parasitized clutches and re-nest, rather than selectively removing parasitic eggs (Spottiswoode & Koorevaar 2012, Spottiswoode 2013). This is a very costly form of defence if the host makes a rejection error, as it then loses its entire clutch rather than just a single misidentified egg. Little Bee-eaters breed in dark subterranean tunnels, so the main cues available to them are likely to be tactile (Spottiswoode *et al*. 2011). The high costs of rejection errors might favour hosts that integrate multiple cues before deciding that a clutch is parasitized and rejecting it.

We tested the hypothesis that Little Bee-eaters require additional cues of parasitism to trigger clutch rejection, such as the sight of an adult parasite (Guigueno & Sealy 2011). We used dummy presentations of adult Greater Honeyguides at the nest with and without the addition of experimental eggs to test whether a combination of these cues is required for Little Bee-eaters successfully to defend themselves against parasitism. We predicted that Little Bee-eaters would preferentially mob Greater Honeyguide dummies rather than non-parasitic controls, and that they would be more likely to reject a clutch if presented with both a Honeyguide dummy and a foreign egg than when presented with either cue alone.

## Methods

Our experiments took place near Choma, southern Zambia, in a *c*. 35-km^2^ area centred on 16°45′S, 26°54′E, during September–November 2013. Greater Honeyguide parasitism occurs throughout our study area. Little Bee-eaters nest in burrows dug into the side of either sandy banks or Aardvark *Orycteropus afer* holes, and Greater Honeyguides are their only brood parasite. We excavated and reconstructed nest burrows (*n* = 29) at the start of every experiment, in order to count host eggs and record their developmental stage.

Our experiment consisted of two treatments applied in a two-by-two factorial design, yielding four treatments in total. We added a foreign egg to 17 nests, whereas 12 nests received no addition. We added eggs to unparasitized clutches shortly before or after clutch completion (one to five eggs, mean = 2.9) to simulate laying Honeyguides which do not remove host eggs, and lay at any point during host incubation (Spottiswoode & Koorevaar 2012). Following previous experiments, for ethical and logistical reasons we used Emerald-spotted Wood Dove *Turtur chalcospilos* eggs to simulate parasitic eggs. This species re-nests readily, is abundant at our study site, and its eggs resemble Greater Honeyguide eggs in size, shape and lack of maculation (Spottiswoode 2013). Following reconstruction of the nest burrow, we presented host parents with a taxidermic mount of either a female Greater Honeyguide or, as a control, a non-parasitic Black-collared Barbet *Lybius torquatus*, which belongs to a related family within the Piciformes, is similar in size to a female Greater Honeyguide (53 g vs. 46 g) and is also common in our study area. Two specimens of each species were alternated, and all four mounts were of birds that had died naturally (flew into windows or killed by bees). We suspended dummies from a wire hoop placed just above the burrow entrance (supplementary Videos S1 and S2), allowing them to move in the breeze. We did not fix dummies in place because pilot trials with stationary dummies often failed to attract the attention of Bee-eaters, and because both parasitic and control dummies should be equally affected by wind movement. We observed nests from > 8 m and recorded the latency to arrival within 5 m of the nest entrance. We noted whether the dummy was mobbed and, if it was, the latency from arrival to mobbing. We defined mobbing as repeated diving flights towards a dummy, accompanied by alarm calls not heard in any other context (Video S1). If mobbing occurred, we removed the dummy after 2 min to prevent damage, and ended the trial. If no mobbing had occurred 15 min after a host bird was seen within 5 m of the nest, we ended the trial. If no Little Bee-eaters approached within 5 m of the dummy for > 15 min, we discarded the trial as they may not have seen the dummy, and repeated it the next day. We checked for egg or clutch rejection by returning to nests 24 and 48 h later and searching for intact or broken eggs lying below the entrance to the nest burrow (Spottiswoode 2013).

We used a Fisher exact test to compare the proportion of each dummy type that was mobbed. As none of the Barbet dummies were mobbed, we restricted further analysis of the data to trials involving Honeyguide dummies only. For this analysis we included two additional nests at which, owing to limited availability of naturally unparasitized nests, we presented a Honeyguide dummy > 48 h after a Barbet dummy. We used a generalized linear model with a binomial distribution and logit link function to test for any effect of the following potential predictors, using backward elimination from a maximal model to optimize AIC: number of host eggs, incubation stage, dummy ID, time of day and latency to arrival within 5 m of the dummy. For variables in the final model, we report effect sizes as odds ratios (OR) calculated from regression coefficients (Nakagawa & Cuthill 2007).

## Results

In support of our first prediction, Little Bee-eater parents mobbed nearly half (10 of 19 trials; Table 1; Video S1) of the Honeyguide dummies, but never mobbed Barbet dummies (none of 10 trials; *P* = 0.005; Video S2). However, contrary to our second prediction, only one trial was followed by rejection of the foreign egg, and this was after the presentation of a Barbet (which did not induce mobbing). This low incidence of rejection resembles that found in a previous study providing only foreign eggs and not models of adult Honeyguides (Spottiswoode 2013).

**Table 1:** Experimental treatments and recorded variables, showing nest ID, number of Little Bee-eater host eggs, incubation stage (scored following Spottiswoode & Colebrook-Robjent 2007), species of dummy used (Black-collared Barbet *Lybius torquatus* or Greater Honeyguide *Indicator indicator*), dummy ID, addition of an Emerald-spotted Wood Dove *Turtur chalcospilos* egg, the time of day at which we started excavating a Little Bee-eater nest prior to carrying out a trial, the time before at least one Little Bee-eater approached within 5 m of the dummy, latency to the start of mobbing, total mobbing duration, total trial duration, the presence of mobbing, whether the clutch was rejected within 48 h of the experiment, and whether the Little Bee-eater pair had been exposed previously to a Barbet dummy

Date	Nest ID	No. of host eggs	Incubation stage	Dummy species	Dummy ID	Dove egg?	Start time (24-h clock)	Time to 5 m (s)	Latency to mob (s)	Mobbing duration (s)	Trial duration (s)	Mobbing?	Rejection?	Re-used?
26/09/2013	MP06	5	1.5	Honeyguide	H2	N	11:00	50	0	120	120	Y	N	N
27/09/2013	MP04	3	0	Barbet	B1	N	14:30	140	NA	0	900	N	N	N
27/09/2013	MP10	5	1	Barbet	B1	N	09:50	65	NA	0	900	N	N	N
30/09/2013	MP16	1	0	Honeyguide	H2	N	11:30	1080	0	120	120	Y	N	N
30/09/2013	MP18	1	0	Honeyguide	H2	N	10:00	450	780	120	900	Y	N	N
02/10/2013	MP23	4	2.5	Barbet	B1	N	16:50	110	NA	0	900	N	N	N
04/10/2013	MP17	1	0	Honeyguide	H2	Y	09:40	145	NA	0	900	N	N	N
04/10/2013	MP22	1	0	Honeyguide	H2	Y	10:45	2660	NA	0	900	N	N	N
04/10/2013	MP24	4	2	Honeyguide	H2	Y	15:00	145	372	10	492	Y	N	N
04/10/2013	MP27	3	0	Honeyguide	H2	Y	16:00	2760	NA	0	900	N	N	N
05/10/2013	MP36	1	0	Honeyguide	H2	Y	08:30	1610	NA	0	900	N	N	N
06/10/2013	MP37	4	5	Honeyguide	H2	Y	08:20	80	855	120	975	Y	N	N
06/10/2013	MP38	4	2	Honeyguide	H2	Y	09:50	0	560	120	680	Y	N	N
07/10/2013	MP39	4	3.5	Barbet	B2	Y	11:50	175	NA	0	900	N	N	N
08/10/2013	MP40	3	0	Honeyguide	H2	Y	08:20	330	NA	0	900	N	N	N
08/10/2013	MP41	2	0	Barbet	B1	Y	11:40	900	NA	0	900	N	N	N
08/10/2013	MP42	1	0	Barbet	B2	Y	16:00	320	NA	0	900	N	N	N
09/10/2013	MP43	4	0	Honeyguide	H2	Y	14:15	640	25	120	145	Y	N	N
10/10/2013	MP44	1	0	Honeyguide	H2	Y	12:15	490	NA	0	900	N	N	N
26/10/2013	MP16a	3	0.5	Honeyguide	H2	Y	08:30	445	155	120	275	Y	N	N
28/10/2013	MP56	3	0	Honeyguide	H2	N	10:15	805	15	120	135	Y	N	N
02/11/2013	MP53	2	0	Honeyguide	H2	N	14:20	2440	NA	0	900	N	N	N
02/11/2013	MP62	2	0	Honeyguide	H2	N	07:15	700	NA	0	900	N	N	N
03/11/2013	MP59	2	2	Honeyguide	H2	N	10:25	270	NA	0	900	N	N	N
03/11/2013	MP68	4	4	Honeyguide	H2	N	11:25	305	50	120	170	Y	N	N
05/11/2013	MP53	4	0	Barbet	B2	N	07:50	995	NA	0	900	N	N	N
05/11/2013	MP59	2	2.5	Barbet	B2	N	13:55	0	NA	0	900	N	N	N
05/11/2013	MP69	2	0	Barbet	B2	N	07:40	1250	NA	0	900	N	N	Y
05/11/2013	MP70	4	0	Barbet	B1	Y	11:15	660	NA	0	900	N	N	Y
06/11/2013	MP68	4	4.5	Barbet	B2	Y	11:40	305	NA	0	900	N	N	N
07/11/2013	MP69	3	0	Honeyguide	H2	Y	09:50	456	NA	0	900	N	N	Y
07/11/2013	MP70	4	0.5	Honeyguide	H2	Y	13:30	860	NA	0	900	N	N	Y
07/11/2013	MP71	3	1.5	Barbet	B1	Y	14:35	1195	NA	0	900	N	N	N
08/11/2013	MP56	3	3	Barbet	B2	N	16:20	95	NA	0	900	N	N	N
13/11/2013	MP72	4	4.5	Barbet	B2	Y	08:35	29	NA	0	900	N	Y	N

We then considered only trials involving a Honeyguide dummy (*n* = 21), to assess potential additional predictors of mobbing. The final model retained two predictors: number of host eggs (*z* = 2.09, *P* = 0.04, OR 3.04, 95%CI: 1.25–10.98) and dummy ID (*z* = –1.47, *P* = 0.14, OR 0.16, 95%CI: 0.007–1.40).

## Discussion

Field observations show that Little Bee-eaters frequently reject or desert entire clutches of eggs in response to Greater Honeyguide visitation (Spottiswoode & Koorevaar 2012). In the present study, we assessed how Little Bee-eaters detect that they have been parasitized, as addition of a foreign egg alone is an insufficient cue to cause clutch rejection (Spottiswoode 2013). Given the particularly high costs of rejection errors in this system, we predicted that Bee-eater parents might require an additional cue, in the form of mobbing a Honeyguide, to trigger clutch rejection. Little Bee-eaters that had laid more eggs at the time of model presentation were significantly more likely to mob Honeyguide dummies, suggesting that either vigilance or incubation attentiveness may increase with the reproductive value of the clutch. However, over half of host pairs did not mob Honeyguide dummies. This is unlikely to be explained by a failure to notice the dummy, as we waited until the Bee-eaters had a clear view of the dummy (were within 5 m) before starting a trial. Bee-eaters frequently flew or perched as close as 0.5 m from a dummy, but appeared to behave as nonchalantly as they invariably did to the control Barbet dummies. We speculate that some were naïve individuals that had no previous experience of honeyguides, as learning is important in acquiring recognition of adult parasites in other brood-parasitic systems (Thorogood & Davies 2012, Feeney & Langmore 2013). There is no reason to believe that Bee-eaters avoided mobbing Honeyguide dummies because Greater Honeyguides mimic predators (Thorogood & Davies 2013a).

Even more unexpectedly, we found that neither the presence of a dummy Honeyguide nor the addition of a foreign egg to the nest, either separately or in combination, caused the Bee-eaters to reject or desert their clutches. Thus, even at those Bee-eater nests where the dummy Honeyguide was mobbed and where a foreign egg was also added to the clutch, host parents still failed to respond. We suggest four potential explanations for this apparent lack of defence against brood parasitism.

First, our experimental set-up may have been insufficiently realistic to mimic the presence of a real Greater Honeyguide. This seems unlikely for two reasons: many previous studies of mobbing behaviour as a defence against brood parasitism have found strong effects of taxidermic dummies similar to those used here, suggesting that in most systems such dummies are an adequately realistic cue (Welbergen & Davies 2009, Feeney & Langmore 2013, Gloag *et al*. 2013).

Secondly, for ethical reasons our experiment did not test any potential effect of punctured eggs, which might provide Bee-eaters with an additional cue of parasitism, as the majority of parasitized clutches contain at least one punctured egg (Spottiswoode & Colebrook-Robjent 2007, Spottiswoode & Koorevaar 2012). As punctured eggs typically rot, they may generate olfactory (Soler *et al*. 2014) as well as tactile cues for parasitism. Field observations suggest that punctured eggs alone are an insufficient cue to stimulate egg rejection, since Little Bee-eaters commonly incubate clutches containing heavily punctured and consequently very rotten eggs (Spottiswoode & Colebrook-Robjent 2007, Spottiswoode & Koorevaar 2012), but it is possible that in Bee-eaters this cue is integrated with others (such as the sight of an adult parasite) to trigger rejection, and that this also improves with experience.

Thirdly, hosts might adjust their defences in relation to the perceived probability of parasitism, as assessed for example by parasite density in the wider environment (Thorogood & Davies 2013b). This could potentially account for the discrepancy between our limited pilot study in 2010 and the present results. The rate of Honeyguide visitation (as assessed by the presence of a Honeyguide egg or punctured host eggs) was lower in the current study year (40.4% in 2013 vs. 56.5% in 2010; *n* = 57 and 62 nests followed to clutch completion, respectively; Fisher exact test, *P* = 0.098), suggesting that parasite density could help to account for this puzzling difference.

Finally, our results could indicate that this Little Bee-eater population is simply poorly adapted in its ability to defend itself against a virulent brood parasite. The failure of Little Bee-eaters reliably to mob honeyguide dummies or to cut their losses and start a new clutch in response to simulated parasitism could explain why this species is the most common Greater Honeyguide host in our study area. However, such hypotheses of evolutionary lag are notoriously difficult to falsify (Kilner & Langmore 2011).

## SUPPORTING INFORMATION

**Video S1**. Little Bee-eaters mobbing a Greater Honeyguide dummy.

**Video S2**. Little Bee-eaters not mobbing a Black-collared Barbet dummy.
